# Extraembryonic Mesenchymal Stromal/Stem Cells in Liver Diseases: A Critical Revision of Promising Advanced Therapy Medicinal Products

**DOI:** 10.3390/cells11071074

**Published:** 2022-03-23

**Authors:** Mohammad Amin Shahrbaf, Masoumeh Nouri, Morteza Zarrabi, Roberto Gramignoli, Massoud Vosough

**Affiliations:** 1 Department of Regenerative Medicine, Cell Science Research Center, Royan Institute for Stem Cell Biology and Technology, ACECR, Tehran 16635-148, Iran; m.a.shahrbaf@sbmu.ac.ir (M.A.S.); m.zarrabi@rsct.ir (M.Z.); 2 Research and Development Department, Royan Stem Cell Technology Co., Tehran 16635-148, Iran; m.nouri@rsct.ir; 3 Department of Laboratory Medicine, Division of Pathology, Karolinska Institute, 141 52 Stockholm, Sweden; 4 Clinical Pathology and Cancer Diagnosis Unit, Karolinksa University Hospital, 141 57 Huddinge, Sweden; 5 Experimental Cancer Medicine, Institution for Laboratory Medicine, Karolinska Institute, 171 77 Stockholm, Sweden

**Keywords:** extraembryonic membranes, umbilical cord, liver cell therapy, amniotic membrane, mesenchymal stem cells, liver regeneration

## Abstract

Liver disorders have been increasing globally in recent years. These diseases are associated with high morbidity and mortality rates and impose high care costs on the health system. Acute liver failure, chronic and congenital liver diseases, as well as hepatocellular carcinoma have been limitedly treated by whole organ transplantation so far. But novel treatments for liver disorders using cell-based approaches have emerged in recent years. Extra-embryonic tissues, including umbilical cord, amnion membrane, and chorion plate, contain multipotent stem cells. The pre-sent manuscript discusses potential application of extraembryonic mesenchymal stromal/stem cells, focusing on the management of liver diseases. Extra-embryonic MSC are characterized by robust and constitutive anti-inflammatory and anti-fibrotic properties, indicating as therapeutic agents for inflammatory conditions such as liver fibrosis or advanced cirrhosis, as well as chronic inflammatory settings or deranged immune responses.

## 1. Introduction

There are many types of liver disease, accounting for a high burden of global diseases [1]. Liver diseases are currently a challenging issue for global public health, considering their high incidence rates, prevalence, and serious morbidities [2]. During the past several years, an increasing trend in hospitalization for chronic liver diseases has been described, particularly for end-stage cirrhosis and fatty liver disease [3]. The burden of chronic liver disease is significant in morbidity, mortality, loss of productive years, and consuming health care resources [4]. Several etiologies—including infection, alcoholism, chemical and biochemical toxins, and malignancies—are exacerbating hepatic conditions [5]. In addition, chronic liver disorders may progress in end-stage liver disease (ELD), with liver transplantation as the only effective treatment for selected patients [6]. Novel therapeutic options, including cell-based therapies and nanomedicine strategies, have been gaining attention as bridge or alternative/adjunct treatment to orthotopic liver transplantation and supporting outcomes for congenital and chronic disorders [7,8].

Cell-based therapies have attracted attention as promising therapeutic option, mainly due to reduced invasive clinical procedures and high regenerative potential [9]. Several stem/stromal cell sources have been proposed and tested preclinically and clinically during the past three decades, but undoubtedly any other multipotent cells have not attracted attention and driven so many clinical trials as mesenchymal stromal cells (MSC). 

## 2. Mesenchymal Stromal/Stem Cells

MSC are certainly the most diffused and largely variable cell products generated and described during the past decades in cell-based therapy approaches. These cells have been defined and collected under a large umbrella of acronyms and nomenclature. The first report describing MSC as a population of fibroblast-like, colony-forming cells was in 1966 by Dr. Friedenstein et al. [10], but MSC existence was postulated 100 years earlier, by Dr. Cohnheim, a german pathologist [11]. More than 30 years ago, Dr. Caplan provocatively coined the term “mesenchymal stem cells”, in response to their multipotency and highly proliferative capacity [12]. However, several years later, he publicly admitted the importance to refine such multipotent cells as stromal medicinal products, whose paracrine action rather than differentiation capacity leads to regeneration induction. 

The exact mechanism(s) of action for different MSC, including extraembryonic MSC, is still largely unknown, but it is well accepted as they home in sites of injury or paracrinely induce regenerative effects through secreted bioactive factors and trophic mediators. Indeed, leading experts have highlighted that patient’s own tissue-resident progenitor cells are the real fabricator for new tissue, supported and enhanced by MSC secreted bioactive factors [13]. Nomenclature changed officially and has been consolidated by the International Society for Cellular Therapy (ISCT) in 2019, 15 years after delineating the release criteria to identify and release MSC products [14]. These cells have been defined “mesenchymal” since the mesenchyme cells are defined as a type of tissue surrounded by a large extracellular matrix (ECM) and characterized by loose intercellular adhesion and lack of polarity. They have been called “stromal” cells as well since, as any structural component in a connective tissue, fibroblastic cells do adhere to culture-treated plastic and expand massively.

The first registered clinical trial where autologous MSC were isolated from bone marrow (BM) and lately re-implanted when in remission by hematological malignancies, is dated 1995 [15]. Since then, the number of clinical applications and registered trials where autologous or allogeneic human MSC have been implanted or infused in patients with different acute or chronic disorders has been growing exponentially and led health economic reassessment. Human MSC have been identified and successfully isolated from several, if not all, adult tissues, particularly BM and adipose tissue as the major sources of clinical-grade cells [16]. The isolation of adult MSCs may be an invasive procedure, not free from side effects and risk for the donor or patient, and frequently associated with low cell yield. The application of non-invasive techniques to isolate MSCs has been reported safe and effective when extraembryonic tissues have been used as cell sources. High quality and more potent MSCs have been successfully derived from perinatal tissue, upon pregnancy termination, with no additional risk for the mother or newborn baby [17], and long-term cryostorage is preferable and undeniably advantageous for regenerative medicine [18]. 

The isolation and purification process of extraembryonic MSCs can be accomplished by the mechanical disruption of the original tissue (i.e., mincing into small fragments) followed by prolonged exposure to different enzymatic solutions, with trypsin or collagenase as the most common and effective tissue dissociation mediators [19]. The extraction and purification of perinatal MSCs have been initially described and validated in human umbilical cord at the time of birth, where authors reported the isolation of CD45 negative cell elements [20]. The use of static cell membrane proteins has been largely described and used to validate hematopoietic elements. Similarly, human MSCs have been reported as lacking characteristic surface proteins such as CD45 or CD34, but constitutively expressing endoglin (CD105) or membrane bound nucleotidase (CD73), in addition to glycoproteins as CD90 or intercellular adhesion molecule (CD44) when grown in in vitro conditions [14].

Scientific literature overflows with reports and studies describing properties and characteristics of human MSCs, including their long-term proliferative capacity in 2D culture conditions, as well as critical ability to differentiate into mesoderm linage—such as bone, cartilage, and fat in permissive conditions—both in vivo and in vitro [21].

## 3. Extraembryonic MSC

The past decade has seen an explosion of experimental and preclinical projects directed toward better understanding of the mechanisms by which MSC act during rescue and repair of injured organs and tissues. Such a plethora of studies on MSC isolated from different tissues have been suffering from different, and frequently fluctuating, methods of isolation and expansion in vitro. Such unpredictable results were even exacerbated by inconsistent and frequently poorly effective maturation protocols performed ex vivo, aimed to generate different somatic cells, including liver cells. Over the years, several researchers and biotech companies have proposed MSCs as multipotent alternative solution to tissue-resident progenitor liver cells, bounded by genetic and epigenetic instabilities, but even more, limited by hepatic maturation level and bio-synthetic activities [22]. 

**Figure 1:**
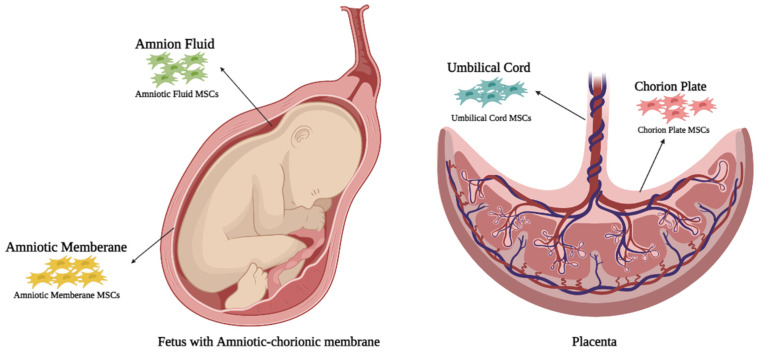
The extraembryonic tissues used for MSCs isolation. Amniotic membrane and amniotic fluid, located in the inner parts of the amniotic-chorionic membrane, are promising sources of MSCs. The placenta and the umbilical cord are the other potential sources of extraembryonic MSCs.

Extraembryonic tissues are frequently described as perinatal tissues or as widely known placenta. Different parts of the human placenta can serve as sources of high-quality MSCs, including amniotic fluid, amnion membrane, chorion plate, the inner cytotrophoblast, outer syncytiotrophoblast and chorionic, yolk sac, umbilical cord tissue, and cord blood (Figure 1) [23,24]. 

The multipotent stem cells derived from extraembryonic tissues are largely acknowledged as valuable sources of MSCs rewarded by safety, accessibility, genome stability, non-tumorigenicity, and rescued/null ethical or religious issues for clinical application compared to the pluripotent stem cells—i.e., embryonic stem cells and induced pluripotent cells [25]. Several characteristics and functional properties have supported the use of MSCs in disparate clinical settings, including their robust potential of immunomodulatory capacities, reduced immunogenicity, differentiation into mesodermal linages, their self-renewal, and proliferative capacity (both in vivo and in vitro) [26]. Here below, we will describe in detail extraembryonic stem/stromal cells that can be isolated from human perinatal tissues, with particular attention to mesenchymal stromal cells of fetal origin. Extraembryonic MSCs role in preclinical or clinical application to reverse or correct liver disorders will be highlighted and discussed in a separate paragraph.

### 3.1. Amniotic Fluid

Amniotic fluid (AF) is an essential ingredient of amnion sac, crucial for the fetus’ growth, development, and protection [27]. Such fluid is commonly harvested by amniocentesis at 15–20th week of the gestation for early diagnosis of the fetal genetic abnormalities [28] or can be collected during caesarian surgical procedure at the end of pregnancy [29]. Over the past several years, purification procedures and cytological descriptions have described the presence of cells floating in AF, primarily of fetal origin (due to skin or intestine exfoliation) or excreted within the fetal urine [30]. Amniotic fluid cells are classified into three main categories based on their morphological and biomedical activity, including the epithelioid type, AF type, and fibroblastic type cells [31]. Epithelioid types are derived from the skin and urine of the fetus and indicate a round shape and slow-growing properties. AF-type cells originated from the placenta and are associated with estrogen, human chorionic gonadotropin (hCG), and progesterone production, and fibroblast-like cells descended from mesenchymal tissue with no hormonal activity and a spindle-shaped morphology [32].

Amniotic liquid is still considered an important source of MSC for cell-based therapy [33]. AF-MSC have been largely described and fulfilling all the criteria needed (surface markers and gene expressions) to be defined as multipotent MSC [34,35,36]. AF-MSCs are accessible and easily isolated/purified in a less invasive manner than other extraembryonic and somatic MSCs. However, the accessibility of these cells is associated with some concerns: first, AF-MSCs can be obtained both at mid-term through amniocentesis and at full-term delivery. However, amniocentesis procedure is considered not free from risk for the fetus and the mother; thus, such a diagnostic procedure is going to be rapidly substituted by less invasive biomolecular and serological analysis. The harvest of AF-MSC at the end of pregnancy has been shown to be attainable, but limited to caesarian section [37]. Such a practice ideally does not introduce any risk either for the newborn or the mother, but it is still poorly offered since the priority is commonly given to the baby and mother, limiting quality and quantity of AF devoted to cell purification.

Functional analysis of AF-MSC, in comparison with other sources of mesenchymal cells, proved expressions of ECM remodeling genes and adhesive factors; secretion of growth and anti-inflammatory factors have also been measured at the same level as somatic MSCs, while the expression of prostaglandins and oxytocin receptors are much higher in AF-MSC [38]. AF-MSCs proliferative capacity has also been shown at higher levels in AF-MSC, as well as their engraftment and adhesion efficiency. The same study also highlighted the reduced immunogenicity of AF-MSCs in comparison to other fetal or adult MSC [39]. Furthermore, AF-MSCs possess important ability to adapt against genotoxic stress, replicative senescence. These fetal-derived MSC have proved superior potential for DNA repair in comparison with adult bone-marrow MSCs, encouraging their application in innovative clinical setting [40]. 

The clinical efficacy of AF-MSC has been mainly ascribed to their paracrine effects, such as secretion of soluble mediators (i.e., TGFβ1 and IL-10), trophic mediators, or angiogenic factors. AF-MSCs are capable to secrete soluble proteases (matrix metalloproteinases (MMP)-2, -9, and -14) responsible for ECM remodeling and fibrosis reversal [41,42,43,44]. AF-MSC effects have been proved in preclinical and clinical settings, where such allogeneic cells have been offered regenerative effects for cardiovascular, renal, musculo-skeletal, gastrointestinal, hematopoietic, respiratory, neurological, and urinary diseases [45]. Furthermore, immunomodulatory and anti-oxidative effects of AF-MSCs have also been reported and described in different regenerative medicine applications. 

### 3.2. Amniotic Membrane

The human amnion or amniotic membrane (AM) is an avascular tissue, characterized in histological analysis by a thick stroma with embedded scarce MSC, while on the surface in direct contact with the fetus, epithelial cells line the surface [46]. Amnion epithelial cells (AECs) originate from epiblasts, during the second week of gestation, before gastrulation and attachment to the uterus; amnion MSC (AMSC) rise from the primitive streak of the trophectoderm, after the three germ layers have been originated [47]. 

Mechano-enzymatic procedures have a proven effectiveness in isolating human AMSCs from full-term amniotic membrane [48]. Freshly isolated or cryopreserved human AMSCs have been reported to express somehow stemness genes such as octamer binding transcription factor (Oct)-3/4, SRY (sex-determining region Y)-box (SOX)-2, Myc, Rex-1, and Nanog in addition to the angiogenic genes *PECAM-1*, *bFGF*, and *VEGF.* Such an expression pattern has been described decreasing during serial passages, and limited to cells at the early passage in vitro [49,50]. 

Flow cytometric analysis on human AMSC confirmed constitutive expression of surface antigens widely accepted as identity markers for MSCs (CD73, CD90, CD105) [14]. The absence of surface markers such as CD31, CD34, CD45, CD106 supports hAMSC identity and homogeneity [51]. Besides, AMSCs express human leukocyte antigen (HLA) class Ia, but lack class II (HLA-DR) [52]. These surface molecules and ectoenzymes are critical mediators to grant AMSC tolerogeneity in allogeneic settings. Human AMSC modulate activation and proliferation of host immune cells, such as T and B cells, or natural killer (NK) cells. Furthermore, extraembryonic AMSCs modulate the production of pro-inflammatory cytokines such as interferon-gamma (IFN-γ), tumor necrosis factor-α (TNF-α), and interleukin (IL)-1β, IL-5, IL-6, IL-9, IL-13, IL-17A, and IL-22 by the innate and adaptive immune cells [53].

Amnion-derived MSCs have also been confirmed as multipotent cells, capable of differentiating into adipocytes, osteoblasts, and chondrocytes [54]. The administration of AMSCs has been described as supportive and beneficial in treating neurological, cardiovascular, and gastrointestinal disorders, but also helpful in a few cancers [55]. 

### 3.3. Chorionic Plate

Chorionic plate MSC (CP-MSC) can be isolated from the chorionic layer of the human placenta by exposing tissue to enzymatic activity. CP-MSC possess similar properties to other extraembryonic or adult MSCs, including the ability for self-renewal and mesoderm differentiation, in addition to “classical” identity proven by selective surface markers [56,57]. The CP-MSC proliferative rate has been described superior to the afore described AMSC [58]. Furthermore, chorionic MSC present enhanced adipogenic potential [59], described as superior to other extraembryonic MSC such as AMSC (whose osteogenic potential is instead reported preferable) [60] or umbilical-cord-derived MSC (prevalently chondrogenic) [61]. Several preclinical studies have described CP-MSC differentiation in to neuronal, pancreatic, angiogenic, and cardiomyocyte-like cells [62].

Recent reports highlighted notable immunomodulatory properties possessed by CP-MSC and peculiar gene expressions and differentiation capacity [63]. CP-MSC have been shown particularly active in reducing T-cell proliferation and IFN-γ secretion [64]. Additionally, it has been reported that CP-MSC secrete high levels of IL-10 and TGFβ1 [65]. 

### 3.4. Umbilical Cord

The umbilical cord (UC) is a multi-layer tissue, characterized by a thick stroma with embedded blood vessels. Human UC consist of two arteries, and one vein enclosed by a gelatinous material called Wharton’s Jelly [66]. Several cells can be isolated from full-term UC, including the most described and commonly used hematopoietic stem cells floating in the umbilical cord blood [67]. Once cord blood is drained out, selective and consecutive enzymatic digestions may facilitate isolation of MSC from the Wharton’s Jelly (WJ-MSC) [68], endothelial cells from the umbilical vein (UVEC) [69], and umbilical cord perivascular cells (UCPVC) [70], and very small embryonic-like stem cells [71]. UC-MSC can be enzymatically isolated from Wharton’s Jelly, perivascular tissue, and umbilical membrane [72,73]. Both natural delivery and caesarean section birth have been offering quality tissues for UC-MSC manufacturing. UC-MSCs are multipotent MSCs, and as all the other somatic or extraembryonic MSCs, present characteristic morphology, plastic adherence, and certain surface markers [74,75]. Notably, gene expression analysis in human UC-MSC resulted in highly angiogenetic and neurogenic pattern profiles compared to other adult MSCs [76]. 

Human UC-MSCs have been applied in regenerative models where they enhanced innate repair capacity, induced secretion of anti-inflammatory cytokines, modulated recipient’s immune recognition and rejection, and inhibited tissue apoptosis as indicated by increased Bcl-xl/Bax protein ratio and decreased cleaved caspase 3 levels [77,78]. 

## 4. Extraembryonic MSC in Clinical Trials

The exponential growth in clinical use and commercialization of primary MSCs has been enhanced by the ethical concerns on pluripotent stem cells (such as embryonic stem cells) and rendered more attractive by past US administration prohibition in ESC study and transplant. The re-designation of MSC as stromal rather than stem cells and their refinement in paracrine properties and features did not affect their use in preclinical and clinical settings.

Considering the technical feasibility, isolation easiness, and accessible tissue, it does not surprise as extraembryonic cells and extraembryonic MSC, in particular, have been largely implemented in disparate clinical trials in previous years. The high level of heterogeneity of MSC is second only to their multiple therapeutic potency and disparate mechanisms of action. Stable and reproducible clinical outcomes require homogenic cell products, both in terms of cellular identity and potency. Nevertheless, high level of heterogeneity has been widely described as a result of different isolation methods or ex vivo cell expansion [79,80]. 

Approximately 1000 clinical trials have been registered up to 2021 [81], using both autologous and allogeneic MSCs, for treatment of a plethora of human diseases and medical conditions. Within these 1000 MSC clinical studies, 179 studies have been conducted using (allogeneic) extraembryonic MSC (excluding umbilical cord blood cells), between 2010 and 2020. Almost 9/10 of such registered clinical trials have been conducted using UC-MSCs. Anti-inflammatory, angiogenic, and trophic effects have been called as the main impact and strength of donor cells. Multipotent differentiation capacity characteristic of primary and expanded MSC have also played an important role in some settings, particularly osteogenic and chondrogenic clinical studies. Two distinct phase 1/2a randomized controlled clinical trials have described therapeutic effects in patients with acute respiratory syndrome post-infection with COVID-19. By injecting UC-derived MSC, the clinical reports described as all anti-inflammatory and immunomodulatory effects have been significantly reduced [82,83,84]. 

## 5. Extraembryonic MSC in Support of Liver Regeneration and Repair

**Figure 2:**
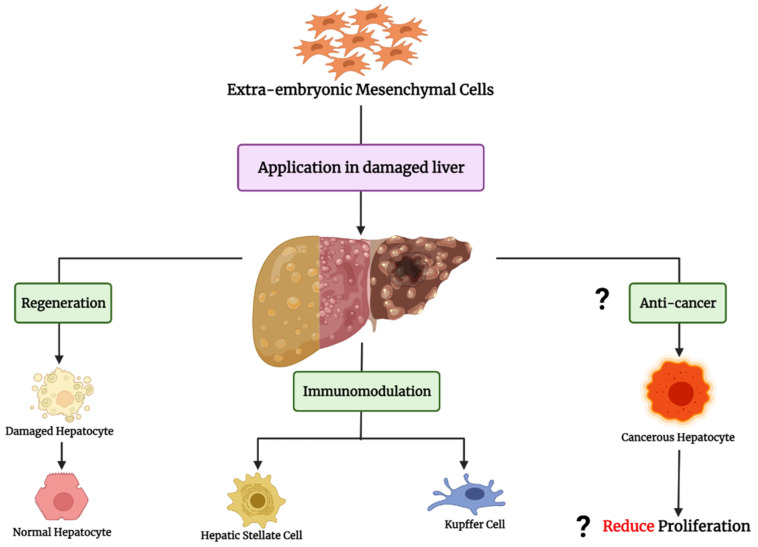
The possible potential application of extra-embryonic MSCs in liver disorders. These cells have high capacity for regeneration induction, immunomodulation (through HSC and Kupffer cell inactivation), and might limit proliferation of cancer cells in the liver tissue.

Extraembryonic MSC may represent innovative and prompt suitable cellular treatments for chronic or congenital liver disorders, if allogeneic MSC work in replacement of damaged/deficient patient’s cells or enhance innate regenerative capacity (Figure 2). 

Extraembryonic MSCs used in interventional medicine or corrective therapy would escalate and remodel current clinical strategies. Many animal studies supported the use of primary extraembryonic cells [85]. However, additional studies are required to fully characterize and profile all the characteristic and functional properties associated with primary extraembryonic MSCs purified from different parts of the perinatal tissues, and many variables can affect their characteristics and efficacy—such as donor genetic background, timing and protocol for collection samples and isolation procedures, and accurate matching between donors and recipients’ needs. Preclinical and quality assessment evaluations are essential and critical for clinical settings, in support of these valuable and easily accessible source of stem cells.

Over the last 20 years, a sparked enthusiasm has been generated for MSC-derived hepatocyte-like cells. Several authors reported the generation of functional hepatocyte-like cells starting from mesenchymal stromal cells, lately resized but not neglected. Embryonic pluripotent stem cells have showed pluripotency, including maturation into (endoderm) hepatocyte-like cells. [86]. Somatic or extraembryonic MSC trans- differentiation into functional and secretive hepatocytes has been largely attempted, initially enthusiastically announced, but lately revised and refined since not completely corroborated by solid measurements. In the early 2000s, high impact reports described MSC-into-Hep maturation both in vitro and in vivo, persuading to clinical applications. Such studies generated results compelled by limited clinical outcome and short-term beneficial effects. Few years ago, an important report highlighted MSC-derived apoptotic bodies as main—if not sole—mediators more than intact viable cells in GvHD treatments [87]. Constitutive expression of adhesion molecules and chemokines, or the ability to respond to soluble chemokines, have been described as diminished or “compromised” when MSC are exposed to immune-reactive or proliferative stimuli [88].

Excitement raised during the first years of the new millennium has been reconfigured when different eminent groups confirmed inefficiency in generating mature and functional hepatocyte-like cells starting from MSC isolated by any somatic or extraembryonic source [89,90,91,92]. Preclinical experiments initially interpreted as direct proof of MSC transdifferentiation into epithelial liver cells were lately corrected by fusion events occurring between donor cells and host hepatocytes. Cell fusion was shown to occur in vivo, as elegantly detailed in preclinical studies using a classical model of fumarylacetoacetate hydrolase (Fah) deficiency [93]. MSC-hepatocyte fusion has been described to be enhanced by the presence of liver injury or chronic disease or in the other disorders later tested [94,95,96]. Then, other groups reconsidered the generation of hepatocytes by hematopoietic cell transdifferentiation, revised in cell fusion events as the mechanism involved [97]. Several revisional studies have illustrated that the generation of hepatocyte-like cells bearing donor markers was not a consequence of MSC maturation into endoderm-like cells, rather as results of cell fusion donor cells are physio-logically prone to [39,98,99]. However, such melting events were not restricted to BM-MSC only, any other types of MSC, including extraembryonic-derived cells, have also been reported to fuse with hepatic parenchymal cells rather than supply new competent cells [100,101]. 

Same as the other adult MSCs, extraembryonic MSCs cells can affect different liver cells—including hepatic parenchymal cells, hepatic stellate cells, and Kupffer cells—by cell-to-cell contact, and paracrine effects [102]. MSC can support hepatocyte’s proliferation and contrast parenchyma apoptosis [103]. On the contrary, donor MSCs can induce apoptosis and contrast activation or proliferation of hepatic stellate cells [104], and liver-resident macrophages (Kupffer cells), inhibiting M1 polarization and promoting production of anti-inflammatory cytokines [105]. However, despite similarities in identity and mechanisms of action among different human extraembryonic MSC isolated from different parts of placenta or amniotic fluid, such cells proved different immunomodulatory and trophic factor production potential, ascribed to different perinatal microenvironments and niches [106].

### 5.1. UC-MSC

UC-MSC can suppress T cell activation in fulminant hepatitis through chitinase 3-like protein 1 (CHI3L1) and NF-κB signaling [107]. Furthermore, the administration of UC-MSC resulted in anti-oxidant (assessed by 2′-7′-dichlorofluorescein diacetate staining) [108], anti-inflammatory, and immunomodulatory effects [109,110]. Hepato-specific gene expression—including plasmatic proteins as albumin, α1 antitrypsin, or α-fetoprotein, as well as phase I mediators (i.e., cytochrome P450 subunits)—was detected in HLC derived from UC-MSC, while scarcely or inconsistently detected in BM-derived HLC [111]. 

Over the past several years, clinical trials have been conducted using UC-MSC in support of hepatocyte transplantation and treatment of metabolic liver disease [112]. In clinical studies on chronic liver disorders, peripheral intravenous administration of UC-MSC was associated with decrement of aminotransferase levels and bilirubin, increase in albumin and MELD score without significant adverse effect [113,114,115,116,117,118,119]. Additionally, they can extend the overall survival rate in long-term follow-up [120]. Furthermore, endurance and extended donor cells have been ascribed to beneficial multiple infusion of UC-MSC rather than single injection [121]. In the context of other non-fibrotic chronic liver disorders, UC-MSC administration in models of non-alcoholic fatty liver disease (NAFLD) and non-alcoholic steatohepatitis (NASH) resulted in hyperglycemia decrement, reversal in transaminase escalation, improvement in lipid profile (such as triglyceride, low-density lipoprotein, or total cholesterol). Improvement in NAFLD/NASH histopathology revealed reduced fat accumulation and oxidative stress in damaged hepatocytes, and reversal in microbiome diversity mediated by toll-like receptor (TLR)-4/NF-κB pathway [122,123,124]. 

### 5.2. AF-MSC

Besides umbilical cells, human primary AF-MSC have also tested as adjunct treatment for liver disorders. Ten years ago, the infusion of 10^6^ rat AF-MSCs—collected between the third to the sixth passage—were administered to fulminant hepatitis model via the portal vein [125]. Such administration resulted in enhanced survival and hepatic functions (low transaminases and high serological albumin levels), reduced cell necrosis, and inflammation. In the same year, another study implanted HLCs derived from syngeneic AF-MSC into acute liver failure (ALF) model of mice, reporting remarkable hepatic engraftment and anti-inflammatory effects [126]. In another study, same amount of AF-MSC injected in a rat model of carbon tetrachloride (CCl4)-induced ALF decreased serological glutamate oxaloacetate and pyruvate transaminase, as well as reduced fibrotic areas, four weeks after cell transplantation [127].

### 5.3. A-MSC

Recent studies observed the positive impact of A-MSC in liver diseases, including reduced inflammation, fibrosis attenuation, and pathological improvement in animal and human models [128,129]. Human A-MSC can attenuate liver damage by paracrine anti-inflammatory cytokines [130], as well as inhibit autophagy by Kupffer mediated by pro-inflammatory mediators [131]. Amnion-derived MSC have proved ability in preventing stellate cell activation [132], as well as enhanced ECM remodeling mediators (i.e., MMP-2, -9, -13, and tissue inhibitor matrix metalloproteinase 1, TIMP-1) leading to fibrosis amelioration [133]. Human A-MSCs have been injected in ALF model, resulting in fibrosis amelioration, and restoring liver function (determined by histopathological and serological analysis) [134,135]. Indeed, it has been reported as intravenous administration of human A-MSC significantly reduced hepatic fibrosis and collagen type-I deposition, prevented recruitment of CD68-positive Kupffer cells and the content of TIMP-1 in the livers in a rat model of ALF [136]. Amniotic MSC have been reported to secrete epidermal growth factor (EGF) and hepatocyte growth factors (HGF), two driving forces in liver regeneration [137,138].

The administration of perinatal A-MSC has been performed also in advanced fibrotic condition, such as chronic liver disorders. The transplantation of A-MSCs in a cirrhotic animal model resulted in remarkable engraftment despite deranged hepatic architecture, probably mediated by MMP-9 and MMP-13, and ameliorated liver microcirculation, reduced inflammation and oxidative stress, with consequent improvement in hepato-specific functions [139,140]. In a recent study, the clinical effects of A-MSC have been reported in a murine model of sclerosing cholangitis, where downregulation of cytokeratin (CK)-19, MMP-9, TNF-α, and monocyte chemoattractant protein-1 (MCP-1) have been described in association with improved pathological score [141]. In addition, A-MSC might affect hepatocellular carcinoma progression. In one study in 2020, it was demonstrated that human A-MSC could migrate to the cancer site and inhibit tumor growth. In addition, these cells express dickkopf-3 (DKK-3), dickkopf-1 (DKK-1), and insulin-like growth factor-binding protein 3 (IGFBP-3), which significantly inhibit the proliferation of cancer cells and increase the apoptosis rate of HepG2 cells. However, further studies are needed to confirm this hypothesis [142].

### 5.4. CP-MSC

In a hepatic context, also CP-MSC have anti-inflammatory, antifibrotic, and high regenerative capacity, helpful for liver disease reversal [143]. Intra-hepatic infusion of CP-MSC in CCl4-injured livers produced ECM remodeling and contained type I collagen and α-smooth muscle actin (α-SMA) expression. On the contrary, the placenta-derived cells supported expressions of albumin and MMP-9 [144]. Furthermore, intrasplenic administration of the same cells reduced the number of both apoptotic (measured by caspase 3/7 activity) and necrotic cells. However, conversely, CP-MSC increased autophagic signals and regeneration capacity, revealed by the light chain 3 II (LC 3II) increment, a marker of autophagy [145].

### 5.5. Exosome Derived from Extraembryonic Cells in Liver Regeneration

Some recent shreds of evidence support the efficacy of UC-MSC derived exosomes as innovative, cell-free liver treatment factor. Intravenous or oral gavage of UC-MSC exosomes may reduce oxidative stress and apoptosis in CCl4 liver injury [146]. In addition, UC-MSC exosomes modulate CD154 expression on liver-resident T cells. The CD154 molecule has been described as an initiative factor of inflammatory response in liver ischemia/reperfusion injury [147,148]. Furthermore, UC-MSC derived exosomes can decrease the collagen type I and III content and reduce transforming growth factor (TGF)-β1 in the liver fibrosis model [149]. In one other study in 2017, extracellular vesicles derived from human embryonic stem cell-MSCs (ES-MSC) significantly ameliorate cirrhosis in thioacetamide-induced chronic liver injury in comparison compared to bone marrow (BM)-MSC and adipose (AD)-MSC. Moreover, ES-MSC expressed anti-inflammatory cytokines and immunomodulatory activities, effective in attenuating liver fibrosis [150].

## 6. Future Perspectives

The summary of the extra embryonic MSCs applications in liver disease is presented in Table 1. The administration of cell-based therapies for liver disease is rapidly progressing. The growing number of registered clinical trials as well as encouraging recent market forecast reports strongly support the practical administration of quality and standardized human extraembryonic stem cells. Among several types of MSC, extraembryonic stromal cells clearly have the benefit of being accessible and non-invasive collection procedure is available. Currently, the application of these extraembryonic multipotent cells in clinical application is mainly limited by standardized manufacturing and quality assessment protocols. Cell transplantation and cellular therapies have long been the realm of university and hospital activities. However, upon validation, the cellular therapies described here could be expanded beyond liver disorders, to the other organs and wider groups of patients.

Extraembryonic stem cells possess multipotency and remarkable immunomodulatory features that may produce a paradigm shift in cell transplantation [151]. Conversely to the other current allogenic cell-based therapies, perinatal MSC do not require immunosuppression nor cause immune-reaction in the recipient. The afore described extraembryonic MSCs actively interact and crosstalk with innate and adaptive immune cells not only by cell-to-cell interactions, but also through paracrine mediators. Such mediators (collectively known as the secretome), composed by both soluble proteins and extracellular vesicles of micro- and nano-size, interact with the target cells and activate endogenous stem and progenitor cells. Both extraembryonic MSCs and their secretome have been described to suppress apoptosis, promote angiogenesis, mediate chemo-attraction, and regulate inflammatory response, stimulate the remodeling of the extracellular matrix and reduce fibrosis. A modern paradigm envisages as perinatal or extraembryonic cells do not necessarily need to mature into adult cell types (i.e., hepatocytes), but they can rescue native parenchymal cells via indirect paracrine mechanisms.

## 7. Conclusions

The plasticity and functional heterogeneity of MSCs may raise potential questions in MSC-based safe and efficacious therapies in the clinical applications. An efficient and substantial proof of differentiation into functional hepatocyte (or cholangiocyte) is the core of all cell transdifferentiation studies. Extra-embryonic MSCs have a high potential for differentiation to mesodermal linage and it is proposed that they can produce HLCs, with similar morphology and physiology to the normal hepatocyte. These cells are also characterized by robust and constitutive anti-inflammatory and anti-fibrotic properties, making them suitable for inflammatory liver conditions such as hepatitis, liver fibrosis, and cirrhosis.

A major challenge in many published studies is validating progenitor/stem cell maturation into functional hepatocytes is the lack of comparison between donor-derived hepatocyte-like cells and real adult human hepatocytes. Hepatic maturation is regularly monitored by the selective upregulation and downregulation of a plethora of genes, proteins, and a few functional assays. Transcriptomic analysis may represent an easy and relatively cheap method to initially test HLCs, but such analysis needs to be performed in direct comparison with primary human hepatocytes. 

## Figures and Tables

**Table 1:** Selected recent preclinical and clinical trials using extra embryonic MSCs in liver diseases.

Ref.	Author/Year	Source of MSC	Type of Liver Disease	Model	Mechanism of Action
[107]	Pan/2021	Umblical cord	Fulminant hepatitis	Mice	Immunemodulatory effects
[108]	Jiang/2018	Umblical cord	Acute liver failure	Mice	Decreased oxidative stress and apoptosis
[109]	Mansour/2019	Umblical cord	Liver fibrosis	Rat	Immunemodulatory effects
[114]	Zhang/2021	Umblical cord	Liver cirrhosis	Human	Improve liver function in short-term follow-up
[120]	Shi/2021	Umblical cord	Liver cirrhosis	Human	Improve liver function in long-term follow-up
[117]	Fang/2018	Umblical cord	Liver cirrhosis	Human	Immunomodulatory effects
[113]	Liang/2017	Umblical cord	Liver cirrhosis	Human	Immunomodulatory effects
[119]	Fang/2016	Umblical cord	Liver cirrhosis	Human	Immunomodulatory effects
[152]	Zhang/2012	Umblical cord	Acute liver failure	Mice	Anti-inflammatory effects
[122]	Li/2019	Umblical cord	Non-alcoholic fatty liver disease	Mice	Increase fatty acid oxidation
[124]	Cheng/2021	Umblical cord	Non-alcoholic fatty liver disease	Mice	Improves lipid metabolism
[125]	Zheng/2012	Amniotic fluid	Fulminant hepatitis	Rat	Engraftment, anti-inflammatory, anti-apoptotic
[126]	Zagoura/2012	Amniotic fluid	Acute liver failure	Mice	Anti-inflammatory effects
[127]	Peng/2014	Amniotic fluid	Liver fibrosis	Mice	Engraftment
[131]	Hua/2019	Amniotic memberane	Acute liver failure	Mice	Immunomodulatory and anti-inflammatory effects
[134]	Lee/2016	Amniotic memberane	Liver fibrosis	Mice	Engraftment, anti-inflammatory, immunemodulatory effects
[136]	Kubo/2015	Amniotic memberane	Liver fibrosis	Rat	Immunomodulatory and anti-inflammatory effects
[139]	Pietrosi/2020	Amniotic memberane	Liver cirrhosis	Rat	Improve hepatic microvascular dysfunction
[140]	Zhang/2011	Amniotic memberane	Liver cirrhosis	Mice	Immunomodulatory and anti-apoptotic effects
[142]	Liu/2020	Amniotic memberane	Hepatocellular carcinoma	Mice	Reduce cell proliferation
[141]	Sugiura/2018	Amniotic memberane	Sclerosing cholangitis	Rat	Anti-inflammatory effects
[144]	Lee/2010	Chorionic plate	Chronic liver failure	Rat	Immunomodulatory and anti-inflammatory effects
[145]	Jung/2013	Chorionic plate	Acute liver failure	Rat	Anti-apoptotic effects, increase autophagy
